# Perceptions of Mobile Apps for Smoking Cessation Among Young People in Community Mental Health Care: Qualitative Study

**DOI:** 10.2196/19860

**Published:** 2020-10-02

**Authors:** Minda A Gowarty, Nathan J Kung, Ashley E Maher, Meghan R Longacre, Mary F Brunette

**Affiliations:** 1 Departments of Internal Medicine and Community and Family Medicine Dartmouth Hitchcock Medical Center Lebanon, NH United States; 2 Geisel School of Medicine at Dartmouth Hanover, NH United States; 3 Center for Technology and Behavioral Health Geisel School of Medicine at Dartmouth Lebanon, NH United States; 4 The Dartmouth Institute for Health Policy and Clinical Practice Geisel School of Medicine at Dartmouth Hanover, NH United States; 5 Department of Psychiatry Dartmouth Hitchcock Medical Center Lebanon, NH United States

**Keywords:** smoking cessation, mHealth, serious mental illness, smartphone application, digital health, psychiatric illness, tobacco treatment

## Abstract

**Background:**

Young adults with serious mental illness are over twice as likely to have tobacco use disorder than those in the general population and are less likely to utilize proven treatment methods during quit attempts. However, little research has evaluated the efficacy of interventions for this group. Smartphone apps may be an underutilized tool for tobacco use disorder among young adults with serious mental illness.

**Objective:**

The aim of this study was to explore attitudes toward smoking cessation apps and preferences regarding app design in young adult smokers with serious mental illness.

**Methods:**

Five focus groups involving 25- to 35-year-old adults with serious mental illness receiving treatment at a community mental health center were conducted between May 2019 and August 2019. Three researchers independently coded transcripts and identified themes using thematic analysis.

**Results:**

Participants (n=22) were individuals who smoke daily: 10 (46%) self-identified as female, 18 (82%) self-identified as White, and 9 (41%) had psychotic disorders. Key themes that emerged included a general interest in using health apps; a desire for apps to provide ongoing motivation during a quit attempt via social support, progress tracking, and rewards; a desire for apps to provide distraction from smoking; concerns about app effectiveness due to a lack of external accountability; and concerns that apps could trigger cravings or smoking behavior by mentioning cigarettes or the act of smoking.

**Conclusions:**

Apps have the potential to support smoking cessation or reduction efforts among young adults with serious mental illness. However, they may require tailoring, optimization, and clinical support to effectively promote cessation in this population.

## Introduction

People with serious mental illness (disabling mood, anxiety, and psychotic disorders) are more likely to develop tobacco use disorders and are less likely to quit smoking than those in the general population, even when using recommended cessation interventions [1-3]. Tobacco smoking is a major contributor to high rates of chronic cardiovascular and lung diseases, high treatment costs (US $160 billion per year in the United States [4]), and to a 10- to 20-year reduction in life expectancy for people with serious mental illness [2,5-7]. While quitting smoking at any age confers health benefits, the harmful health effects of smoking worsen as the number of cigarettes and duration of smoking increase [8]. Intervening at an early age can dramatically reduce the risk of smoking-related disease and can mitigate early mortality [8].

A significant body of literature [9-12] has described unique challenges faced by people with serious mental illness who wish to quit smoking, which include both sociocultural influences (such as lower education and income, as well as higher stress levels) and neurobiological influences (including the modulating effect of nicotine on dopaminergic pathways in schizophrenia). Additionally, people with serious mental illness may endorse using tobacco to manage challenging psychiatric symptoms and to overcome difficulties with socialization [10,12,13]. Furthermore, some cessation medications (eg, bupropion) may not be indicated for a subset of people with serious mental illness [14]. While overall, pharmacologic therapies are safe and effective for people with psychiatric illnesses [3,11,15-17], behavioral interventions are also needed to teach cessation skills, provide education about the safety and efficacy of smoking cessation medications, and support mental health. However, little research has focused on interventions for young adults with serious mental illness and tobacco use disorder, and effective strategies for addressing tobacco use disorder among young adults with serious mental illness have not yet been established.

Research on treatment in the general population is informative. Behavioral therapies for treating tobacco use disorder improve abstinence rates in youth and young adults in the general population, and are recommended by US Clinical Practice Guidelines [18]. A meta-analysis [19] that extracted data for young adult participants (18 to 24 years old) from studies of adult smokers found that behavioral interventions designed for older age groups were also effective for young adults. Research on interventions designed specifically for young adults found the most promising results from telephone and web-based interventions [20]. Yet evidence-based, scalable tobacco use disorder treatment approaches, such as telephone Quitline counseling, are underutilized by young people. For example, only 8.5% of US Quitline callers from 2011 to 2013 were young adults [21], and that percentage decreased to 5% in 2016 [22]. Young adults with and without serious mental illness report frequent quit attempts [23], but they are typically unaided and unsuccessful [24]. In our previous research [25], we found that young adults with serious mental illness, in contrast to middle-aged adults with serious mental illness, were less likely to initiate evidence-based tobacco use disorder treatment after education or standard motivational interviewing, suggesting that more appealing approaches to treatment are needed for this group.

Young people are avid users of smartphone technology [26], and smartphone apps offer beneficial features for behavioral intervention delivery. App content can be accessed on-demand, allowing users to capitalize on fleeting moments of motivation. Additionally, apps can deliver personalized and interactive content, including proactive notifications based on time and location [27]. In a recent survey [28], we found that 80% of young people with serious mental illness used smartphones, similar to the overall rate among young adults with low incomes (in the United States) [26]. Furthermore, 70% of young adults with serious mental illness were willing to try digital health interventions on their device [28], indicating preliminary feasibility for digital interventions for tobacco use disorder treatment in this group.

While smartphone apps offer a number of attributes that seem well-suited to young adults with and without serious mental illness, optimal development and implementation of this technology requires input from the intended users. However, phase I (design and refinement) and phase II (feasibility, proof-of-concept, or pilot testing) trials of smoking cessation apps are often omitted or are not reported in published literature [29]. Assessing end users’ desires regarding app content and features is important in developing appealing interventions as well as for cost-effective implementation and reliable interpretation of effectiveness [29]. Recent work has addressed this gap in knowledge for middle aged adults with serious mental illness [30-33]. However, to our knowledge, research evaluating perceptions of apps for smoking cessation among young adults with serious mental illness has not been published.

Given the need for improved engagement in tobacco treatment among young adults with serious mental illness, and the promise of smartphone apps as an accessible and tailorable vehicle for behavioral intervention, we sought to explore attitudes among young adults with serious mental illness who smoke toward using apps for smoking cessation as well as preferences regarding app design. The goal of this study was to obtain information that could guide the tailoring of mobile app interventions to the unique needs of this population.

## Methods

### Participants and Recruitment

We used purposive sampling to recruit potentially eligible participants from a single large community mental health center in New England between May 2019 and August 2019. Recruitment occurred via flyers posted in waiting rooms and clinician referral. Eligible participants were 18 to 35 years old, English-speaking, stable in their outpatient mental health treatment for serious mental illness (ie, no hospitalization in past 30 days per chart review), and smoked daily. We chose the age range of 18 to 35 years to focus on adults for whom quitting smoking has the greatest potential long-term mortality benefit. While the National Young Adult Health Survey found that cessation attitudes were similar among 18- to 24-year-old and 25- to 34-year-old adults [34], it also demonstrated that smoking trajectories differ between these age groups [35]. Thus, we stratified patients by age into 2 groups—18 to 24 years and 25 to 35 years—to better characterize participants’ cessation needs. This paper presents findings from the 25 to 35-year-old age group. Patients were excluded if they were pregnant or had a current, unstable substance use disorder (per chart review or per the participant’s mental health center clinician).

Prior to each focus group, research staff read the study information sheet aloud with the participants and discussed the purpose of the study with them. All eligible participants were deemed competent to give consent, which participants provided verbally. Participants received a US $30 gift card to a retail store after completing the focus group. The New Hampshire State Institutional Review Board approved and monitored all study activities.

### Procedures

#### Brief Survey

Participants completed a 10-item survey prior to the start of focus group discussions. The survey included questions about the participants’ gender identity, race, tobacco use, and technology use. Technology use included questions about app use, including whether participants had ever downloaded a health-related app (such as a step tracker or stress management app). Participants’ DSM-5 (Diagnostic and Statistical Manual of Mental Disorders, Fifth Edition) psychiatric diagnoses, as determined by their mental health center clinicians, were gathered by chart review.

#### Focus Groups

We conducted 5 focus groups between May 2019 and August 2019, each of which included 3 to 6 participants and lasted approximately one hour. A researcher trained in qualitative methods (MG) moderated the focus groups, with a second member of the research team present to take field notes. Focus groups took place at the community mental health center where participants received services.

The moderator posed questions in a funnel-type structure [36], starting with broad questions about participant experiences with smoking, narrowing to experiences with quitting or reducing smoking, and finally to perceptions about using smartphone apps to quit or reduce smoking. We followed a semistructured format, using probing and clarifying questions to elicit thick descriptions of participants’ perceptions and experiences. The focus group discussions were audiorecorded and transcribed. A member of the research team who was present at the focus groups compared the transcriptions to the audio files to ensure accuracy. Focus groups were conducted until thematic saturation was reached, which was identified when previously recognized themes repeated without the emergence of new themes [37,38]. This occurred after the fifth focus group. The focus group discussion guide can be found in Multimedia Appendix 1.

### Data Analysis

Transcripts were iteratively analyzed using thematic analytic techniques [39]. Three researchers (MG, NK, and AM) independently coded each transcript using ATLAS.ti (version 8, ATLAS.ti Scientific Software Development GmbH). After conducting an immersive review of the data set, an initial set of codes was generated using a deductive-inductive approach. This approach allowed us to generate codes based on prior empirical data on facilitators and barriers to quitting smoking, while also allowing new codes to emerge from the data set. The 3 researchers met regularly to refine the code definitions until reaching a final code structure, which they each independently applied to the entire data set. Additional meetings were held to discuss discrepancies in applying the final codes to the data set until consensus was reached through negotiation [40]. Memoing was used throughout the coding process to facilitate a deeper understanding of how the codes relate to each other, and how codes could be represented by unifying themes [41]. The themes were then developed into thematic statements, and emblematic quotations where chosen to illustrate how the themes developed from the data. Negative case analysis was used to ensure the entire data set was represented in the emerging themes.

## Results

### Study Participants

Participants (n=22) were individuals who smoked daily and who were stable in their community mental health treatment. Almost half of participants (10/22, 46%) self-identified as female, most (18/22, 82%) self-identified as white, and 9/22 (41%) were diagnosed with psychotic disorders. Technology use data are presented in Table 1.

**Table 1:** Focus group participant characteristics.

Characteristic			Value, n (%)
**Demographic and clinical characteristics (n=22)**			
	**Gender**		
		Male	12 (54)
		Female	10 (46)
	**Race**		
		White	18 (82)
		Mixed	3 (14)
		I don't know	1 (5)
	Psychotic disorder diagnosis		9 (41)
**Technology use characteristics (n=19^a^)**			
	Use internet ≥ twice daily		18 (95)
	Ever downloaded an app		18 (95)
	Ever downloaded a health app		16 (84)
	Would try an app if recommended by a doctor		15 (79)

^a^Technology use data were missing for 3 participants.

### Focus Group Themes

#### Facilitators and Barriers to Quitting Smoking

During the initial discussions about reasons for smoking and participants’ prior experiences with quitting, a number of themes emerged regarding facilitators and barriers to quitting. Reasons for quitting included the desire to save money and concern regarding their children’s, pets’, and personal health. For example, one participant stated:

I feel like whenever I quit smoking, I can smell things easier, I can taste things more, and I can breathe better.

Group 1 Participant 3

Commonly cited reasons to continue smoking despite a desire to quit included addiction to nicotine, smoking as routine, and smoking to manage mental health symptoms (such as stress, anxiety, and depression):

...since I have bipolar, it helps. Smoking helps me not have so much anxiety, but when I do quit, I get even more.

Group 5 Participant 1

Relapse triggers included socializing with other smokers, smelling tobacco smoke, and seeing people smoke on television or in movies. Of note, while many participants mentioned prior use of nicotine replacement therapy or cessation medications such as varenicline or bupropion with varying degrees of success, most stated that either they or their clinicians were not comfortable with using prescription cessation medications due to concern for psychiatric side effects. For example:

I took Chantix for a while...it really did help. I was smoking like two packs a day, and then when I was on the Chantix I was smoking maybe six cigarettes a day...and a doctor told me that it might contribute to depression. And I decided to get off it because I already have depression, and...I don’t want to worsen it.

Group 4 Participant 1

That’s what I was told. None of my doctors, nobody will give me Chantix. Because of how bad I am.

Group 4 Participant 2

#### Role of Apps in Supporting a Quit or Reduction Attempt

Focus group discussions regarding apps included participants’ prior experiences with health-related apps as well as their perceptions regarding the role of smoking cessation apps. Their prior experiences were generally positive, though with variable effect on behavior. As participants discussed mobile apps for smoking cessation, many expressed interest in using apps during a quit attempt and described a number of ways that apps could offer support, such as providing motivation during a quit attempt, increasing awareness of smoking habits and money spent, and providing distraction from smoking. They also described app limitations, such as lack of external accountability and the potential to trigger cravings. These themes, along with illustrative quotations, are summarized in Table 2. Subthemes that emerged from these discussions are subsequenty presented.

**Table 2:** Major themes regarding app use for smoking cessation, with corresponding illustrative quotations.

Themes		Illustrative quotation	
**Suggested ways apps could support a quit attempt**			
	Providing ongoing motivation		“I think it’d be cool if you can be able to, like, challenge someone else who was trying to quit smoking, but like a buddy, right? But it’s through the app. And they could be thousands of miles away, but you got that one person...” [Group 5 Participant 2]
	Increasing awareness of smoking patterns		“Tracking when you smoke, the times you smoke, how much a day you smoke, what led you to smoke that much. Like, I feel like those are all helpful things to know” [Group 4 Participant 1]
	Providing distraction		“Yeah, ‘cause when you’re having a craving, you just look at it [the app] and maybe it’ll tell you, like, uh, go for an hour run, or you know, tell you some sort of structure to keep your mind off of you smoking. Something to keep you busy, keep your hands busy...” [Group 3 Participant 1]
**App limitations in supporting a quit attempt**			
	Lack of external accountability		“...how is this going to know when I’m smoking a cigarette or not? I can just say I’m not...and then I’ll be sitting there smoking a cig, you know” [Group 2 Participant 4]
	Possible triggers		“You’d have to use, like a code word for cigarette so people don’t think it in their heads because once they think ‘cigarette,’ they’re more likely to smoke” [Group 2 Participant 3]

### Prior Experiences With Apps

Many participants described interest in health-related apps such as fitness trackers or mood trackers but noted varying degrees of benefit during their prior experiences with these types of apps. Participants discussed using the apps to review information about their personal habits (ie, their “stats”), but altering a behavior based on this feedback was rare. For example, a participant using a step tracker said,

It was cool, a cool thing. It didn’t really matter because I was working every day and I’d get about the same amount of steps every day.

Group 1 Participant 4

Only one participant described increasing her activity level to achieve goals in her step tracker; others simply reviewed their steps without this leading to change in behavior. Another participant found a symptom tracker useful to bring to her clinicians so they could change her treatment plan but did not utilize the information herself.

### Motivation

Participants felt that while motivation to quit is a prerequisite for using a smoking cessation app, the app would need to provide features that could facilitate ongoing motivation. Participants suggested a number of ways that apps could motivate them, such as receiving support from other people within the app, feedback about progress (such as cigarettes avoided or money saved), and rewards such as financial incentives or badges. They also mentioned the importance of distraction to avoid cigarettes and suggested that an app could offer distraction by providing suggestions for alternate activities instead of smoking or by including games within the app.

### Social Support

While one participant was concerned that an app would lead to missed social interactions and reduced social support for quitting, others discussed desire for the app to incorporate social support. Multiple participants suggested the ability to connect with other smokers for support within the app. One person suggested a chat feature:

So I think if you could, like, message someone on the app that’s using it at the same time...if you could communicate with someone else using it, like chat with them...

Group 3 Participant 5

Other participants recommended group challenges, similar to those seen in popular fitness apps:

I think it’d be cool if you can be able to, like, challenge someone else who was trying to quit smoking, but like a buddy, right? But it’s through the app. And they could be thousands of miles away, but you got that one person...

Group 5 Participant 2

While most of the participants who valued social support in their quit attempt mentioned other smokers or peers, one suggested involving family members as part of the quit plan in the app:

Or someone, a family member or a loved one, could work with the app and you could earn points or something like that by doing that more than smoking.

Group 2 Participant 3

### Tracking Progress

A prominent theme across focus groups was the desire to track the number of cigarettes smoked per day. Many participants wanted to track cigarettes to increase their awareness of how much they smoke. Others added that they would like to record additional information about their smoking so they could learn their typical smoking patterns:

Tracking when you smoke, the times you smoke, how much a day you smoke, what led you to smoke that much. Like, I feel like those are all helpful things to know.

Group 4 Participant 1

Some participants noted that quitting all at once can feel insurmountable, but suggested that tracking cigarettes could be motivating by allowing them to see progress toward quitting:

Maybe not to quit, because to me, quitting is not realistic. If I could reduce, like, instead of smoking 20 cigarettes, if I could smoke 5 cigarettes a day, that would’ve been a big difference.

Group 2 Participant 1

Most participants cited money as a major motivator to quit or reduce their smoking and would want the app to provide a feature that tracks money spent on cigarettes or money saved by avoiding cigarettes:

I should, I feel like what’d probably help me, if like, if I kept track to see how much I’m paying, spending on them [cigarettes] because... if I kept track to see how much I’m paying every time, I’d be like, ‘Okay, that needs to stop.’ Because that’s money that I could be using.

Group 4 Participant 3

### Skills Training

While tracking was a common theme, fewer participants suggested skill-based features for behavioral change. One participant recommended that an app include

Something to disassociate the triggers and patterns associated with smoking.

Group 1 Participant 5

Another stated that

The app has to be informative. It should have tips on how to reduce the urge to smoke.

Group 2 Participant 1

However, such statements were far less common than recommendations for tracking.

### Rewards

A common theme across focus groups was the desire for rewards within the app. Some participants mentioned financial rewards such as gift cards for using the apps while others recommended that the app award badges for progress, based on their experience with other health behavior change apps:

I think that those badges, you know, when I get those rewards...nobody else sees them. I’m the only one, but it is a reward to myself...It’s just mine. Nobody can take it from me. This is stuff that I worked hard for, and worked, probably, pretty hard for.

Group 5 Participant 2

### Distraction

Another prominently mentioned feature across focus groups was the benefit of distraction during a quit attempt. Many participants described using distraction as a tool for avoiding cigarettes during prior quit attempts and felt that a smartphone app would be well-suited to providing distraction. Some participants suggested that the app could provide games to play to avoid smoking. Others felt that the app could provide suggestions for alternative activities to smoking:

Yeah, ‘cause when you’re having a craving, you just look at it [the app] and maybe it’ll tell you, like, uh, go for an hour run, or you know, tell you some sort of structure to keep your mind off of you smoking. Something to keep you busy, keep your hands busy...

Group 3 Participant 1

### App Limitations

Most participants felt that the biggest barrier to using an app to quit or reduce smoking was a lack of motivation to change smoking behavior in general, and that once motivated, there were few barriers other than practical issues related to any mobile technology (eg, limited phone battery, inability to use phone if lost or broken, limited cellular or wireless internet service in certain locations). However, two main concerns arose regarding the limitations of apps during a quit attempt—a lack of external accountability and the potential to trigger cravings.

A prominent theme across focus groups was the need for external accountability during a quit attempt and concern that an app would not be able to provide this. Related to their discussion of the importance of monitoring progress, participants voiced concerns about the temptation to report false information to an app. One participant, who had previously used a cigarette tracking app, noted:

I tried a cigarette counter once, and realized it wasn’t gonna work to help me quit smoking because I, at that time, I would cheat and not log all of my cigarettes. I don’t even know why I cheated because it’s not like anyone was watching.

Group 1 Participant 2

In another focus group, a similar sentiment arose:

...how is this going to know when I’m smoking a cigarette or not? I can just say I’m not...and then I’ll be sitting there smoking a cig, you know.

Group 2 Participant 4

Other participants felt that apps might have limited benefit without a method of confirming smoking status. One participant stated:

Like, the phone has to have a contact system where you’re, like, Face-Timing somebody who’s coming to visit you to go meet for coffee and then they’re smelling you and they can see if you smoked any cigarettes by doing that test...

Group 2 Participant 3

Despite general agreement that entering information into the app on an honor system is a limitation, participants who mentioned biochemical verification as a means to achieve this did so unfavorably. In one group, a participant described mistrust of the accuracy of breath carbon monoxide monitoring. In another focus group, a participant who mentioned concern about false reporting to the app went on to consider breath carbon monoxide monitoring, but then immediately discounted it, saying,

...that’s a little much...

Group 2 Participant 4

Participants made few recommendations about features that should be avoided in a smoking cessation app, but a common concern was the possibility that the app could trigger cravings. Based on their prior experiences with smoking triggers, they were concerned that any mention of cigarettes in the app could increase their desire to smoke:

You’d have to use, like a code word for cigarette so people don’t think it in their heads because once they think ‘cigarette,’ they’re more likely to smoke.

Group 2 Participant 3

## Discussion

### Principal Results and Comparison With Prior Work

In this study, we explored the attitudes of young adults with serious mental illness who smoke toward quitting smoking and the use of apps for this purpose. Overall, our findings share significant overlap with those of prior early phase trials in middle-aged adults with serious mental illness [30,33]. We found that young adults with serious mental illness who smoke shared similar reasons for quitting or continuing to smoke as those in other populations and were interested in using apps during a quit or reduction attempt. Participants voiced a desire for apps to provide ongoing motivational content during a quit attempt, features to increase awareness of smoking habits and money spent, content that could be used as a distraction from smoking, reward features, and social support for quitting smoking. They also described potential app limitations, such as the temptation to enter false information into the tracking features, and an app’s potential to trigger cravings. This group’s concern about psychiatric side effects of cessation pharmacotherapy is an additional important characteristic that could be countered or monitored with digital technology.

Our analysis revealed a tension between participants’ desire to see progress via tracking features and their fear of seeing personal failure via the same features. Similar to qualitative findings in studies of middle-aged adults with and without mental illness [30,33,42-44], participants in our study voiced a strong desire for cigarette- and money-tracking features that could demonstrate progress and enhance motivation during a quit attempt. However, participants also described using other apps with similar features that had not affected their patterns of behavior. Additionally, and similar to other adults with and without mental health issues [33,45], they expressed concern that recording information that suggests a lack or loss of progress might be demotivating and could lead to relapse or to the temptation to enter false information into the app. Although tracking smoking and viewing progress were popular features noted by this study group and those in other research [30,42,43,46], in one study of general population adult daily smokers [43], these features were not associated with improved abstinence.

Similar to other studies of adults with serious mental illness, in general [10,12,13,33], and young people with mental health conditions, in particular [47], participants described their main barriers to quitting as inability to resist cravings, using smoking to manage mental health symptoms, and smoking out of habit. Yet, only a few suggested that apps could teach strategies to overcome these barriers. Instead, most participants conveyed the common perception that it just takes “will power” to quit, rather than the application of skills to tolerate stress and urges without smoking. Skills training, such as advice on changing routines and improving coping strategies for cravings, has been shown to improve abstinence outcomes for both in-person [48] and app-based [43] interventions. Taken together, these data suggest that tracking features are appealing and may enhance engagement or motivation, but other skill building or clinical support features for cessation skills would need to be prominent and engaging to ensure use within the apps.

Many participants in this study recommended that cessation apps include financial rewards or badges awarded by the app for progress. There is a growing body of literature to support the use of rewards in the form of praise [48] and financial incentives [49,50] for smoking cessation. Inclusion of rewards features in smoking cessation apps, therefore, has promise to improve both engagement and abstinence outcomes.

While prior studies assessing users’ desire for specific features in smoking cessation apps demonstrate mixed results regarding the role of social support for quitting within apps [33,42,44,45], participants in our study clearly valued this feature. In line with prior findings regarding the role of social influence on smoking behavior in people with serious mental illness [10,51], our participants highlighted the importance of the effects of social environment and peer interaction on their smoking behaviors and described the potential benefit of an alternative smoke-free support system within the app. They specifically voiced a desire for peer support through chat functions and competitions, as opposed to smoking cessation coaching or technology coaching that has been described in other studies [30,32]. This desire for peer support is similar to preferences described by young adults with serious mental illness in a study [52] assessing the possible role of digital support for mental health diagnoses and highlights the perceived importance of peer-to-peer interaction for overcoming the stigma and challenges associated with smoking as well as living with mental illness. Although one study [53] demonstrated the potential of a motivational intervention delivered by peers, Dickerson and colleagues [54] described numerous challenges to cessation associated with a peer mentoring approach for people with serious mental illness. While this feature may improve engagement in the app and promote overall well-being, its effect on smoking behaviors requires further study.

The landscape of smoking cessation apps is rapidly changing. Currently available apps are variable in their content and features [27,29,55-58], and few contain content that adheres to clinical practice guidelines [27,55-57,59-61]. A recent literature review [29] found that most smoking cessation apps included self-tracking features, but only one-third included social support and one-third included rewards systems. Furthermore, most apps included three or fewer features, which typically involved education, tracking, and a variable third feature [29]. Another recent review [58] found that less than half of available apps included advice on changing routines or coping with cravings. Apps that include high-quality, evidence-based content as well as an array of both desired features and features previously found to be effective should be tested in this population.

Compared to other populations of smokers, the young adults with serious mental illness in this study voiced similar reasons for smoking, quitting smoking, and relapsing after a quit attempt [34,62,63], but they expressed specific concerns regarding medication safety in relation to their mental health conditions. A wide body of evidence has demonstrated the safety and efficacy of cessation pharmacotherapy in the setting of mental illness [3,11,15-17]. Thus, improving utilization of these treatments can substantially impact quit rates. Our findings suggest that content in a smoking cessation app tailored for young adults with serious mental illness can likely overlap substantially with content in other smoking cessation apps, with additional information about medication safety for people with mental illness.

### Limitations

Several study limitations merit consideration. First, participants were not required to be interested in quitting smoking in order to participate in the study; individuals who are actively engaged in an attempt to quit smoking may ultimately prefer different app features. Second, participants were predominantly White residents in a small New England city, and may not be representative of smokers with serious mental illness who have other demographic characteristics or are from other geographic regions. Yet the substantial overlap between our findings and those of research in other populations [30,33,42,43] supports the validity and generalizability of the themes conveyed here. Lastly, most participants had not previously used apps for smoking cessation, and therefore, relied on anticipated future desires for their responses. While anticipation of future desires can be subject to a number of biases, assessing participants’ preferences prior to exposure to specific apps is important to understanding how they will appraise apps during initial use. Future work should assess responses to cessation apps in this population.

### Conclusions

Young adults with serious mental illness expressed similar reasons for quitting smoking or continuing to smoke compared to those expressed by other populations of individuals who smoke, but low cessation treatment utilization rates and quit rates suggest that other treatment modalities are needed. Apps have the potential to support quit or reduction attempts for young adults with serious mental illness in a number of ways, such as providing ongoing motivation during a quit attempt, increasing awareness of smoking habits and money spent, and providing information and support for using cessation skills. However, while young adult with serious mental illness who smoke are interested in using apps, further tailoring, optimization, and clinical support may be necessary to effectively promote cessation in this population.

## Appendix

Multimedia Appendix 1 Focus group discussion guide.
