# Supplementary material for: Perceptions of Mobile Apps for Smoking Cessation Among Young People in Community Mental Health Care: Qualitative Study
Source: JMIR Form Res. 2020 Oct 2;4(10):e19860. doi: 10.2196/19860 (PMC7568217; doi:10.2196/19860)
Supplement: Multimedia Appendix 1 [file formative_v4i10e19860_app1.docx]

## Multimedia Appendix 1. Focus Group Discussion Guide.

Facilitators/Barriers to Quitting

Tell us a little about how you started smoking.

What keeps you smoking now?

Tell us a little about whether you have ever thought about trying to cut down on your smoking, or quit smoking?

What is it about smoking that made or makes you think about cutting down or quitting?

What have you or your friends used to help you try to cut down or quit (if anything)?

What do you think would help people your age cut down or quit smoking?

What would be most important for you and your friends to have to help you cut down or quit smoking?

What gets in the way of people your age cutting down or quitting smoking?

What is your biggest barrier to cutting down or quitting smoking?

Smartphone App for Quitting Smoking

Please tell us about what smartphone apps you have used. What kind of apps do you like to use, and what do you like about them?

What about apps for relaxation?

If you have ever used a smartphone app to help you with health, tell me about that experience. (It could be for either physical health or mental health or relaxation.)

What was the app for?

What was good about it?

What was NOT so good about it?

What parts of a health app make it useful?

If you were talking to someone who makes apps, what would you tell them to include to make the apps helpful?

What would help you or other people your age want to use an app to quit smoking?

What might get in the way of you or people your age using an app to help them quit smoking?

For anyone who would not be interested in using an app to help you quit smoking, talk a little bit about why.

What features should be included in an app that can be used to help people your age quit smoking?

For instance, if you were to say, ‘if X or Y was in the app, that would make it more helpful’

(Eg. tracking, tips to quit, connecting to social media, etc)

What features should NOT be included in an app that can be used to help people your age quit smoking? (What things have you seen in other health apps that you don’t like, or that might get in the way)
